# Physical therapy intervention studies on idiopathic scoliosis-review with the focus on inclusion criteria^1^

**DOI:** 10.1186/1748-7161-7-4

**Published:** 2012-01-25

**Authors:** Hans-Rudolf Weiss

**Affiliations:** 1 Gesundheitsforum Nahetal, Alzeyer Str. 23, D-55457 Gensingen, Germany; 2 Following a paper presented at the 8th annual meeting of the SOSORT, Barcelona 2011: Hans-Rudolf Weiss, MD, Elisabete Santos Leal, OMT, Ulrike Hammelbeck BSc.: Proposal for the SOSORT inclusion criteria for studies on physiotherapy

## Abstract

**Background:**

Studies investigating the outcome of conservative scoliosis treatment differ widely with respect to the inclusion criteria used. This study has been performed to investigate the possibility to find useful inclusion criteria for future prospective studies on physiotherapy (PT).

**Materials and methods:**

A PubMed search for outcome papers on PT was performed in order to detect study designs and inclusion criteria used.

**Results:**

Real outcome papers (start of treatment in immature samples/end results after the end of growth; controlled studies in adults with scoliosis with a follow-up of more than 5 years) have not been found. Some papers investigated mid-term effects of exercises, most were retrospective, few prospective and many included patient samples with questionable treatment indications.

**Conclusion:**

There is no outcome paper on PT in scoliosis with a patient sample at risk for being progressive in adults or in adolescents followed from premenarchial status until skeletal maturity. However, papers on bracing are more frequently found and bracing can be regarded as evidence-based in the conservative management and rehabilitation of idiopathic scoliosis in adolescents.

## Background

Scoliosis is a three dimensional deformity of the spine and trunk, which may deteriorate quickly during periods of rapid growth [1-3]. Although scoliosis may be the expression or a symptom of certain diseases, eg. neuromuscular, congenital, due to certain syndromes or tumors, the majority of the patients with scoliosis (80-90%) are called ‚Idiopathic' because a certain underlying cause still has not been found. The treatment of the symptomatic scoliosis may primarily be determined by the underlying cause. The treatment of the so-called idiopathic scoliosis is determined by the deformity itself. As most of the scoliosis progress during growth, some also in later life, the main aim of any intervention is to stop curvature progression [1,2].

**Figure 1:**
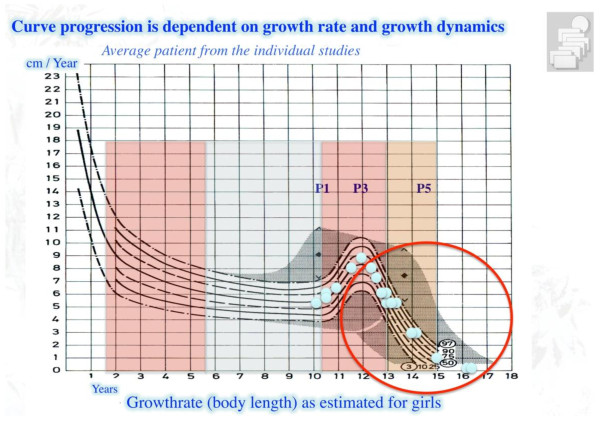
**Growth rate (body length) as estimated for girls**. This figure shows that immature individuals experience two phases of growth with higher velocity. One may be called the baby spurt with a descended characteristic (0 to approx. 6 years of age). The other is the pubertal growth spurt (approx. 10 to 13 years). Between these two phases with higher growth velocity, a flat phase of growth with little risk for progression occurs (Figure modified from Weiss and Weiss 2005). The distribution of the average patients from the studies as presented in Table 1 is demonstrated (blue spots). With kind permission of Pflaum, Munich (Weiss HR: Best practice in conservative scoliosis care. 4th edition in press).

While children grow until they have fully matured, there are certain times with more or less growth during childhood and adolescence and curvature progression is more or less probable during different phases of growth [1,2] (Figure 1). The ‚baby spurt' ends at the age of five and a half to six years followed by a ‚flat phase,' which lasts until the first signs of maturation. With the first signs of breast development or pubic hair, the pubertal growth spurt begins (P1) and in its ascending phase 2/3 of progression may occur [1]. Shortly after the growth peak (P3) menarche in girls/voice change in boys appears to indicate the onset of the descending phase of growth up to its cessation (P5).

**Figure 2:**
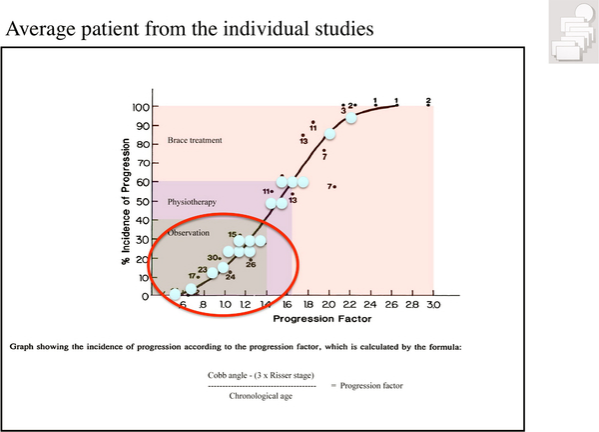
**Incidence (risk) of progression can be calculated according to the formula by Lonstein and Carlson (1984)**. According to the indication guidelines (Weiss et al. 2008) we have to distinguish between an-Indication for observation only (Incidence (risk) of progression-40%),-Indication for physiotherapy (Incidence (risk) of progression 40-60%),-indication for bracing (Incidence (risk) of progression 60% and more); The average patient from the majority of the papers on physical therapy found in Table 1 have no indication for treatment but for observation, only (blue spots). With kind permission of Pflaum, Munich (Weiss HR: Best practice in conservative scoliosis care. 4th edition in press).

In patients with idiopathic scoliosis during adolescence, the risk for being progressive can be calculated using the formula by Lonstein and Carlson [4]. Based on this formula the treatment indications of scoliosis patients during growth are determined [5] (Figure 2). The guidelines derived from this knowledge have been established by leading members of the SOSORT (International Society on Scoliosis Orthopaedic and Rehabilitation Treatment) in order to avoid over- and under- treatment as well. The formula, Cobb angle-(3 × Risser stage) divided by the chronological age is established to calculate the progression factor indicating the treatment indications during growth as demonstrated in the SOSORT guidelines [5].

While premenarchial girls at average have Risser 0, the Risser sign arises after the onset of menarche/voice change (in boys). A 14 year old girl usually has Risser 3, sometimes 4, a 15 year old girl usually has 4, sometimes 5.

A 10 year old girl with 20° and the first signs of maturation before the onset of menarche is usually Risser 0. Therefore, the progression factor is 2, indicating a risk for being progressive of 90%.

A 15 year old girl with 20° usually is 2.6 years postmenarchial with Risser 4. Therefore, the progression factor in this case is 0.53, indicating there is no more risk for being progressive and that there is no more indication for further treatment.

**Figure 3:**
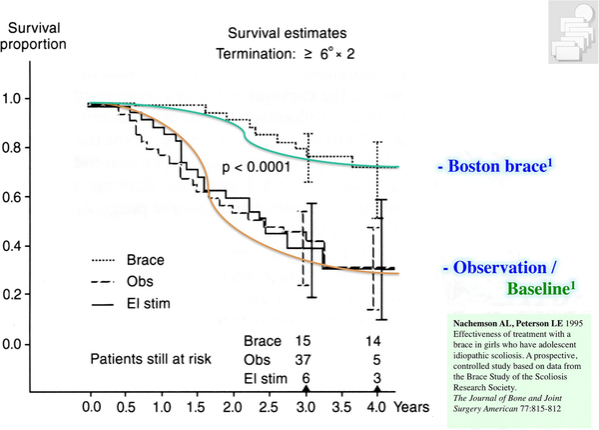
**Graph of the survival analysis as presented by Nachemson and Peterson (1995)**. Per definition every patient being progressive is eliminated from the count of the study and therefore has not survived. At the start of the observation period therefore we have 100% of patients in the study and at the end of the observation period there are 30% left (non progressive) in the observation group and 70% left (non progressive) in the patient group treated with a Boston brace (Figure modified according to Nachemson and Peterson 1995). With kind permission of Pflaum, Munich (Weiss HR: Best practice in conservative scoliosis care. 4th edition in press).

Physiotherapy, corrective bracing and spinal fusion surgery are the treatment modules currently applied in the treatment of scoliosis [6]. While there are prospective controlled studies for the use of the Boston [7,8] (Figure 3) and the Chêneau brace [9] and also one RCT on physiotherapy [10], no papers have been found to support spinal fusion surgery on a higher level [11].

Few prospective controlled studies (Level II) on bracing started in immature patient samples and ended after cessation of growth [7-9] the studies on physiotherapy published so far seem to have variable study designs.

Purpose of this Pub Med review was to analyze the data provided by Pub Med on physiotherapy in patients with scoliosis as well as the materials already presented in systematic reviews [10,12] focusing more closely on the maturity and treatment indication of an average patient from the various studies in order to explore as to whether physiotherapy in the treatment of scoliosis really can be regarded as being evidence based or not?

## Materials and methods

A PubMed (and hand search of papers cited in previous reviews) for outcome papers on PT was performed in order to detect study designs and inclusion criteria used for studies on physiotherapy. Retrospective controlled studies (Level III), prospective controlled studies (Level II) and randomized controlled studies (Level I) with untreated controls having scoliosis were taken into account, but also other study designs were recorded.

The search (October 24th, 2011) was performed for manuscripts using the mesh terms "scoliosis AND physiotherapy/exercises/exercise. The inclusion criteria were as follows:

- Patients: Diagnosis of AIS (Adolescent Idiopathic Scoliosis) in adolescence and adulthood, confirmed through X-rays; we focused on patients in growing age;

- Experimental intervention: patients treated exclusively with physiotherapy, without any other associated intervention;

- Control group: any kind of scoliosis patients with observation only;

- Outcome measures: only Cobb degrees: results could be reported in absolute terms or as percentage of patients improved/worsened;

- Study design: any study design to be able to find hidden controlled trials.

Previously published reviews (10,12) were scrutinized. Targeting at a proper treatment indication the risk for being progressive of the average patient from the studies was calculated according to the formula published by Lonstein and Carlson [4], whenever Cobb angle, Risser stage and age of the average patient was documented.

In case the Risser stage of the average patient was not available, an estimation of the Risser was performed according to the average age of the group using the data available in literature [13-15]. As there is a clear correlation between Risser sign (stage) and chronological age [15] this estimation can be regarded as being meaningful in larger samples, but not necessarily in individual cases.

For adult patients with scoliosis, the focus was laid upon controlled studies with an untreated control group with a follow-up of > 5 years as the slow progression in adulthood will usually not exceed the margins of the technical error of 5° usually calculated for Cobb angle measurements [2].

## Results

193 papers displayed when the terms ‚Idiopathic Scoliosis AND physiotherapy' were entered. 167 papers displayed when the terms ‚Idiopathic Scoliosis AND exercises' were entered and 139 papers displayed when the terms ‚Idiopathic Scoliosis AND exercise' were entered. In the majority of the papers physiotherapy was not the main or only treatment. Many papers were found on bracing, other papers did not account for the Cobb angle as an outcome parameter.

Hand search was performed for studies mentioned in papers, especially on the review papers found [10,12].

Real outcome papers (start of treatment in immature samples/end results after the end of growth) have not been found. Some papers investigated mid-term effects of exercises, most were retrospective, few prospective and many included patient samples with questionable treatment indications.

Most of the studies had patient samples not meeting the treatment indications as proposed within the guidelines (see Table 1). Sample sizes ranged from 9 to 591 with 75% of the samples as described in the various studies exceeding the number of 50 [10].

**Table 1:** Papers on the outcome of physiotherapy in patients with scoliosis sorted by age.

		Age	Cobb degrees	Risk of Progression
**Author**	**Year**	**Average**	**Average**	**Estimated**

Weiss	2003§	10	21	90%

Ducongé	2002	10, 1	15,6	50%

Mollon	1986	10,8	16	50%

Klisic	1985	11	14	35%

Ferraro	1998	11,6	14,9	35%

Rigo	1991	12	19,5	35%

Negrini	2006a	12,4	15,1	15%

Weiss	1997	12,7	27	60%

Weiss	2003$	13	29,5	60%

Mooney	2000	13,1	33,5	85%

Negrini	2006b	13,4	30,9	60%

Stone	1979	13,5	10	0%

den Boer	1999	13,6	26	27%

McIntire	2006	14	29	20%

Otman	2005	14, 1	26,1	5%*

Mamyama	2002	16,3	31,5	25%*

Maruyama	2003a	16,3	33,3	25%*

Weiss	1992	21,6	43	**

As can be seen, only 7 out of 19 samples published at average had a risk of progression exceeding 40% and by this had an indication for treatment (38%). One study had a pre- post design and should be excluded**. Three other papers were with a patient sample that was (nearly) outgrown and would not need any treatment*. The studies by Weiss 2003, Mollon and Rodot 1986 and Ducongé 2002 had a wide range of materials and included also many prepubertal patients not yet at risk. The patient sample from Weiss, Weiss and Petermann (2003) was subdivided into an immature (§) and a more mature sample ($)

(* Patients nearly outgrown/outgrown; ** Patients outgrown/pre-/post intervention study)

Some studies investigated immature patient samples with curvatures of less than 15° not yet in the range of requiring treatment (Figure 1), many of them were already mature at the start of the study, and not needing any treatment at all (Figure 2).

One study (n = 74) compared two different unproven concepts against each other [16].

There was no paper with an adult sample of patients treated by physiotherapy compared to untreated controls.

One study [17], questions the value of non-operative treatment commonly used for adult scoliosis patients. The authors state that documented costs are substantial and no improvement in health status was observed within 2 years.

Two other short-term controlled studies have been published, stating that surgical treatment was superior to conservative treatment [18,19] and claims have been made that these studies are reaching Level II evidence.

Other studies published recently did not study the change of Cobb angle [20-22].

## Discussion

No paper was found concerning patients at risk for being progressive, followed to skeletal maturity under physiotherapy treatment alone. Claims made to regard physiotherapy as an evidence based method of treatment, are therefore, scientifically unjustified [[10,12] Romano et al. Cochrane Review in press].

The only evidence on Level II is found in the immature sample (n = 94) from the prospective controlled study from our group [23]. However, this group of patients was not followed up to skeletal maturity.

**Figure 4:**
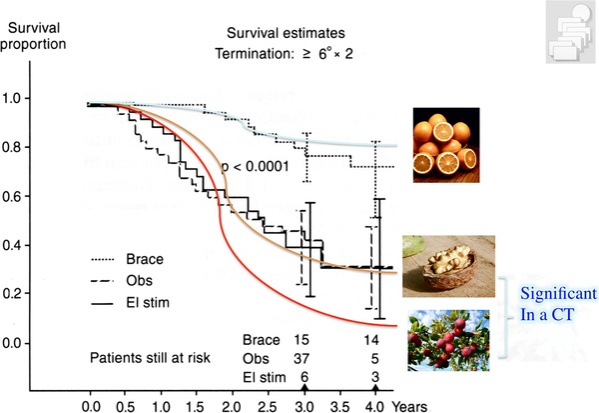
**Fictitious survival analyses explaining why a comparison of two different treatments without an untreated control group does not make sense: When one group of patients undergoing the ‚nuts' treatment does not benefit from this, but is compared to the ‚plums' treatment group increasing the curvature angle, there surely may be differences in controlled studies (randomized or not)**. But it does not show one of the interventions is really beneficial to the patient ('orange'). So, a controlled study design without an untreated control group is not providing any evidence for an intervention as investigated with the help of this study design [as demonstrated in 16]. With kind permission of Pflaum, Munich (Weiss HR: Best practice in conservative scoliosis care. 4th edition in press).

Another paper comparing two unproven groups of physiotherapy (general exercises vs. SEAS [Scientific Exercise Approach to Scoliosis], described in [12] against each other) does not seem to provide any evidence as this study design does not make sense [16] because the differences found between the two groups cannot necessarily be regarded as leading to the conclusion that one of the therapies might be of any benefit to the patients treated:

When one method is not effective and has no better results than observation only, the other method could also lead to deterioration and therefore be statistically different (Figure 4). This is the cause why only controlled studies with an untreated control group can be regarded as a valid source of scientific information.

Nevertheless, the authors claim in their conclusion: *This data confirms the effectiveness of exercises in patients with scoliosis who are at high risk of progression. Compared with non-adapted exercises, a specific and personalized treatment (SEAS) appears to be more effective *[16].

This study included seventy-four consecutive outpatients with adolescent idiopathic scoliosis, mean 15 degrees (standard deviation 6) Cobb angle, 12.4 (standard deviation 2.2) years old, at risk of bracing who had not been treated previously [16].

Italian girls with an average age of 12.4 years surely can be estimated as being postmenarchial with a Risser 1 at least. When calculating the average patient from this sample using the Lonstein and Carlson formula [4,5] with a curvature of 15 degrees one can estimate a risk factor of < 1 and therefore this patient sample is not at risk, but is a benign sample of not needing any treatment because the risk for progression does not exceed (actually is < 15%) 40%, which according to the guidelines would be the indication for physical treatment. But even if the Risser sign is assumed as unlikely to be 0, there was no indication for treatment as the progression factor would still be 1.2, only, thus still below 40%. So also this calculation would lead to the conclusion of clear unnecessary overtreatment and in no way the patient sample is at risk of needing a brace as stated in the paper.

This study is only one example of documented malpractice and unfortunately literature is full of samples not needing any treatment, but claims have been made from these studies that physiotherapy would be of benefit.

The authors have also published a retrospective study [24] including a „worst case analysis "with a patient sample (n = 112) of 13.2 years and a Cobb angle of 23.4 degrees. Also this sample lacks any indication for treatment (***23.4-3 × Risser 2 **(estimated benign, because many Mediterranean girls have Risser 3 at the age of > 13 years, as have German girls too)**/13.2 = 1.32**, which makes less than 40% chance for progression with a clear indication for observation, only) As these girls surely are in the descendent phase, the prognosis is getting better every day and as a curve of < 30° is very unlikely to progress after cessation of growth, there is surely no urgent indication for treatment and no real risk for the need of brace treatment*).

The Chinese RCT [25] has a patient sample (n = 80) with an average age of 15 years at the start of the follow-up period and a follow-up time of 6 months at an average. 15 year old girls (girls are the main population in samples with AIS) usually do not have significant residual growth left and do not necessarily need any treatment. So this study, even with the most important study design (RCT) cannot contribute to the search for evidence for PT in scoliosis (Table 1). Additionally there is no evidence that the curvature does not return to the initial value after the period of physiotherapy.

**Figure 5:**
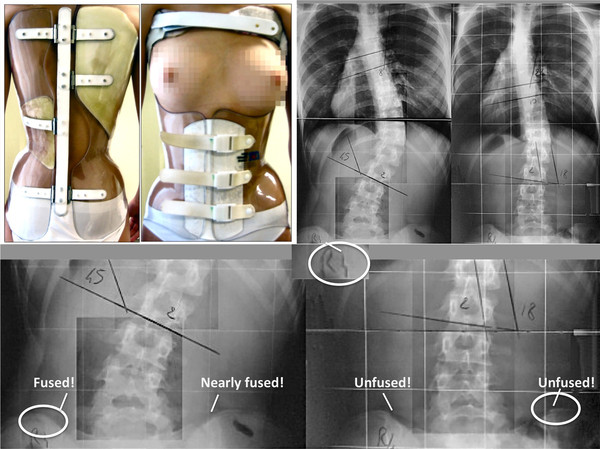
**A sample of figures demonstrating that also in bracing it is not uncommon to treat mature individuals**. The female patient on the upper left clearly is mature (Breast staging: Tanner 5). The x-rays as published within the same figure together with the clinical picture [26] on the first glance seem to show a drastical improvement of the curve as claimed by the authors. First of all, a permanent result like this cannot be achieved in a mature patient and is therefore not credible (on the left side x-ray a Risser 4 was estimated with fusion of the right iliac crest apophysis). Secondly, the Risser sign on the right x-ray seems more immature when compared to the left which could lead to the assumption that the right x-ray was the first and the left one was the last one and by this demonstrates a drastical curve progression. With kind permission of Pflaum, Munich (Weiss HR: Best practice in conservative scoliosis care. 4th edition in press).

The problem of treating mature patients and claiming beneficial outcomes is also evident in bracing [26] (Figure 5).

As can be seen in Table 1, only 7 out of 19 samples of patients published [23-25,27-41] had a risk of progression exceeding 40% and by way of this had an indication for treatment (38%). One study had a short-term pre-post design and should be excluded [27]. Three other papers were with patient samples (n = 80 [25], n = 69 [31], n = 53 [32]) that were (nearly) outgrown and would not need any treatment [25,31,32].

The studies by Weiss 2003, Mollon and Rodot 1986 and Ducongé 2002 [23,29,34] with respect to the materials included were not homogenous, had a wide range of materials and included also many prepubertal patients not yet at risk. Therefore finally only 4 out of the 19 samples [23,35,37,41] can strictly be regarded as having had an indication for physical therapy. However, all these 4 samples were postmenarchial when the observation started at the descendent part of the pubertal growth spurt.

No controlled paper with adult patients at risk for progression (curvatures exceeding 35°) [2] has been found.

According to the findings from this review, studies on physiotherapy in idiopathic scoliosis patients have the following shortcomings:

- *wrong treatment indications*

- *lack of risk for progression*

- *lack of comparability*

- *lack of homogeneity*

Prospective study designs should not be overestimated when the material within the study can be inappropriate [42]. In studies on scoliosis this is the case when mature samples without any treatment indications are studied using prospective controlled or even randomized designs (e.g. [25]). Maybe there is a benefit also for this population from applying PT, but only a patient sample at risk for being progressive in the well renown range of proper treatment indications can be accepted and can contribute to evidence in this field. This may also be the problem within some Cochrane reviews [43].

**Figure 6:**
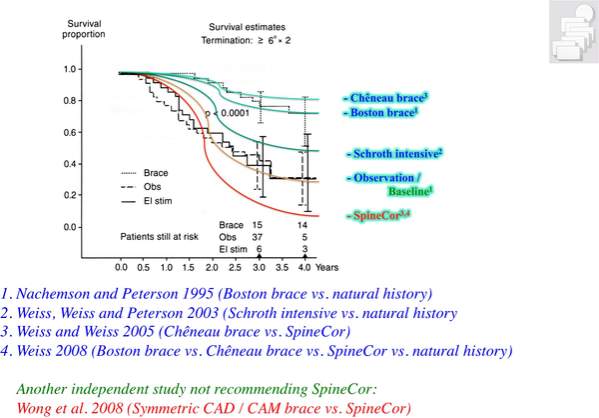
**Synopsis of the survival proportions of the different studies available for comparison**. For the treatment of an immature patient in the pubertal growth spurt the SpineCor seems worthless with a survival rate (8%) of less than observation, only (30%). The immature Schroth (physiotherapy) sample has a survival rate of 50% (estimated from the premature end results as the sample was not followed up to skeletal maturity), the Boston brace 70% and the Chêneau brace of the 1999 standard 80%. As the Schroth sample was not followed up to skeletal maturity (> 30 months only) this graph for physiotherapy is fictitious as it shows a follow-up of 4 years. The other limitation of the Schroth sample is the lack of homogeneity, also including patients not at actual risk. On the other hand, the prospective controlled study on Schroth seems to be the one providing the highest evidence for PT at this stage. With kind permission of Pflaum, Munich (Weiss HR: Best practice in conservative scoliosis care. 4th edition in Press).

It is also important to see the current evidence for physiotherapy during growth within the context of the other module of conservative treatment such as bracing. The only paper presenting at least some evidence for physiotherapy is the prospective controlled paper from my previous working group [23], however, this patient sample was not followed up to the end of growth (maturity). Within this study we find a subsample of patients at higher risk for being progressive. The controls from this study were non progressive in 30% without any treatment which compares well to the controls in the SRS brace study [7]. In immature patients intensive inpatient physiotherapy can halt progression in 50%, however the Boston brace without PT will be effective in 70% [7]. The Chêneau brace of the 1999 standard is effective in 80% [9] while today effectiveness has increased to > 90% [44]. Therefore, bracing seems to be the most important approach in the conservative management of patients with scoliosis during growth (Figure 6).

Recent papers present samples not followed up by x-ray investigations and therefore could not be compared to other studies from this review [20-22]. However, in at least two of these studies [21,22] mature patient samples were studied (and in part treated as in-patients for many weeks), who were not at risk for deterioration.

Thus overtreatment seems to be an important issue in the studies on conservative management of scoliosis.

There are a few studies on conservative treatment of adult scoliosis patients published recently [17-19], but no studies with an untreated control group. Although the limitations of these studies were discussed, the authors draw conclusions even though their studies have major shortcomings. In one of the studies the authors [17] state themselves: An important caveat of this study was that the treatment was not randomized and therefore the treatment group might have deteriorated if not for the treatment they received. Bridwell et al. [18] had drop-out rates of more than 50% in the non-operative group, so no conclusions are justified from this paper, because a ‚worst case' analysis would possibly lead to the opposite conclusions.

A similar paper, published in 1995 [45] was also accepted for publication in the American edition of the Journal of Bone and Joint Surgery, although the conservative sample had a return rate of 50% only. Scientifically these papers do not merit being published as their material is poor and the conclusions drawn, invalid.

Study design may be prospective controlled, however as the problems (complications) of spinal surgery arise after many years, mostly with a lifetime risk of 40-50% [2,46], a follow-up of two years seems very questionable and therefore these papers comparing surgery to conservative treatment of scoliosis seem no valid source of information.

An agreement of the scientific community on common inclusion criteria for future studies on PT seems necessary. We suggest the following: (1) girls only, (2) age 10 to 13 at the first signs of maturation (Tanner II), (3) Risser 0-2, (4) risk for progression 40-60% according to Lonstein and Carlson.

It would be even better to only include patients from the ascendant phase of the pubertal growth spurt: (1) girls only, (2) age 10 to 12 at the first signs of maturation (Tanner II), (3) Risser 0, (4) risk for progression 40-60% according to Lonstein and Carlson.

However, the problem would be that the number of the patients included would be very limited as has been shown in a prospective controlled paper on bracing using this kind of inclusion criteria [9]. Usually scoliosis in adolescent girls is detected after the onset of menarche and therefore the suggestion also including patients with Risser 1 or 2 seems to be more reasonable.

The postural correction plays a major role in physiotherapy like in bracing [7-9,47] treatment and can be achieved for instance by side shift exercises or the recent developments of the Schroth method [31,32,48,49]. The methods for exercising (Yoga, Pilates, SEAS, DOBO MED) presented in the review by Fusco et al. [12] are not sufficiently evaluated and should be questioned.

There is still no evidence that physiotherapy exercises can decrease the progression of scoliosis in immature samples with idiopathic scoliosis (with significant Cobb angles > 15 degrees) and thus the correction by braces is emphasized. However, physiotherapy exercises should be regarded as a complement to bracing concerning postural control during activities of daily living (ADL) [49]. Postural experience and postural correction are important to stimulate a good posture in grown-up individuals. A specific method of teaching the patient to achieve an optimal postural control was introduced by Schroth and optimized recently [49]. The positive effect of physical exercise on peak bone mass and on balance performance/coordination in growing children/adolescents should of course not be underestimated [50,51].

PT may have a beneficial effect on the patient with idiopathic scoliosis as this has been demonstrated in many pre-/post cohort studies [12], however during the most vulnerable period of the pubertal growth spurt PT should never be regarded as the only meaningful mode of treatment [52].

## Conclusions

- Most of the studies included patients not yet or no more at risk for being progressive.

- Additionally, the papers on adults with scoliosis (conservative vs. surgical) have a follow-up period too short to draw any conclusions as complications of surgery in most of the cases appear more than 5 years after surgery.

- There was no outcome paper on PT in patients with idiopathic scoliosis at risk for being progressive followed from the premenarchial status until skeletal maturity. Therefore, only bracing can be regarded as being evidence based in the management of scoliosis patients during growth.

- There is little evidence that PT might have beneficial effects on spinal curvatures. Specific exercises like Side Shift or Schroth could be favoured.

- Future studies on physiotherapy in idiopathic scoliosis should only be accepted when they follow the inclusion criteria as presented within this paper.

## Competing interests

The author is advisor of Koob-Scolitech, Abtweiler, Germany.
